# External validation of a machine learning model to predict hemodynamic instability in intensive care unit

**DOI:** 10.1186/s13054-022-04088-9

**Published:** 2022-07-14

**Authors:** Chiang Dung-Hung, Tian Cong, Jiang Zeyu, Ou-Yang Yu-Shan, Lin Yung-Yan

**Affiliations:** 1 Department of Critical Care Medicine, Taipei Veteran General Hospital, No. 201, Section 2, Shih-Pai Road, Taipei, 11217 Taiwan; 2 School of Medicine, National Yang Ming Chiao Tung University, Taipei, Taiwan; 3 Philips Research China, Shanghai, 200072 China

**Keywords:** Hemodynamic Stability Index, Early prediction model, Machine learning, Clinical decision support, External validation

## Abstract

**Background:**

Early prediction model of hemodynamic instability has the potential to improve the critical care, whereas limited external validation on the generalizability. We aimed to independently validate the Hemodynamic Stability Index (HSI), a multi-parameter machine learning model, in predicting hemodynamic instability in Asian patients.

**Method:**

Hemodynamic instability was marked by using inotropic, vasopressor, significant fluid therapy, and/or blood transfusions. This retrospective study included among 15,967 ICU patients who aged 20 years or older (not included 20 years) and stayed in ICU for more than 6 h admitted to Taipei Veteran General Hospital (TPEVGH) between January 1, 2010, and March 31, 2020, of whom hemodynamic instability occurred in 3053 patients (prevalence = 19%). These patients in unstable group received at least one intervention during their ICU stays, and the HSI score of both stable and unstable group was calculated in every hour before intervention. The model performance was assessed using the area under the receiver operating characteristic curve (AUROC) and was compared to single indicators like systolic blood pressure (SBP) and shock index. The hemodynamic instability alarm was set by selecting optimal threshold with high sensitivity, acceptable specificity, and lead time before intervention was calculated to indicate when patients were firstly identified as high risk of hemodynamic instability.

**Results:**

The AUROC of HSI was 0.76 (95% CI, 0.75–0.77), which performed significantly better than shock Index (0.7; 95% CI, 0.69–0.71) and SBP (0.69; 95% CI, 0.68–0.70). By selecting 0.7 as a threshold, HSI predicted 72% of all 3053 patients who received hemodynamic interventions with 67% in specificity. Time-varying results also showed that HSI score significantly outperformed single indicators even up to 24 h before intervention. And 95% unstable patients can be identified more than 5 h in advance.

**Conclusions:**

The HSI has acceptable discrimination but underestimates the risk of stable patients in predicting the onset of hemodynamic instability in an external cohort.

**Supplementary Information:**

The online version contains supplementary material available at 10.1186/s13054-022-04088-9.

## Introduction

Hemodynamic instability is a crucial and common condition in intensive care unit (ICU). One-third of ICU patients will develop hemodynamic instability and receive hemodynamic interventions with a mortality rate of 40–59% [1, 2]. The diagnosis of hemodynamic instability manifests in a variety of clinical parameters, including vital signs, physical examination, and laboratory measurements which reflect underlying pathophysiology of cardiovascular system, impaired tissue perfusion, and cellular metabolism [1, 3]. Timely diagnosis and early initiation of intervention are still challenging, since the most important information for clinical decision is diluted by large quantities of data on comprehensive hemodynamic parameters from time to time.

Early warning score of hemodynamic instability has the potential to improve the timely detection and then initiation of intervention [4, 5]. Single-parameter shock indicators such as systolic blood pressure (SBP) and shock index (heart rate/SBP) were reported to detect hemodynamic instability [6, 7]. However, it either deteriorated in later stage of shock or underestimated the risk by only addressing cardiovascular system changes. Machine learning (ML) models with multi-parameters were developed as another way to continuously monitor and identify patients at high risk of hemodynamic instability [8–10]. It was reported that Hemodynamic Stability Index (HSI), which was developed based on patients’ data from US cohort across 54 hospitals, significantly outperformed single parameters like SBP and shock index with good generalization in another US cohort—MIMIC III [9, 11]. Thirty-three variables were selected as input features of the machine learning model which included vital signs, laboratory and blood gas measurements, and ventilation settings.

External validation is critical to quantify the generalizability of a risk prediction model, whereas only 5–7.1% of published studies were externally validated [12, 13], even less in the cohort from different regions and clinical practices by independent researchers. Performance drift of prediction model occurred frequently in external validation due to the difference in outcome incidence, heterogeneity of predictors’ effect, and difference in case mix, i.e., the distribution of predictors values [12]. In this study, we aim to independently validate the above-mentioned HSI model in an external cohort from different region and clinical practice and to evaluate the case-mix effect on the performance drift.

## Methods

### Cohort selection

**Fig. 1:**
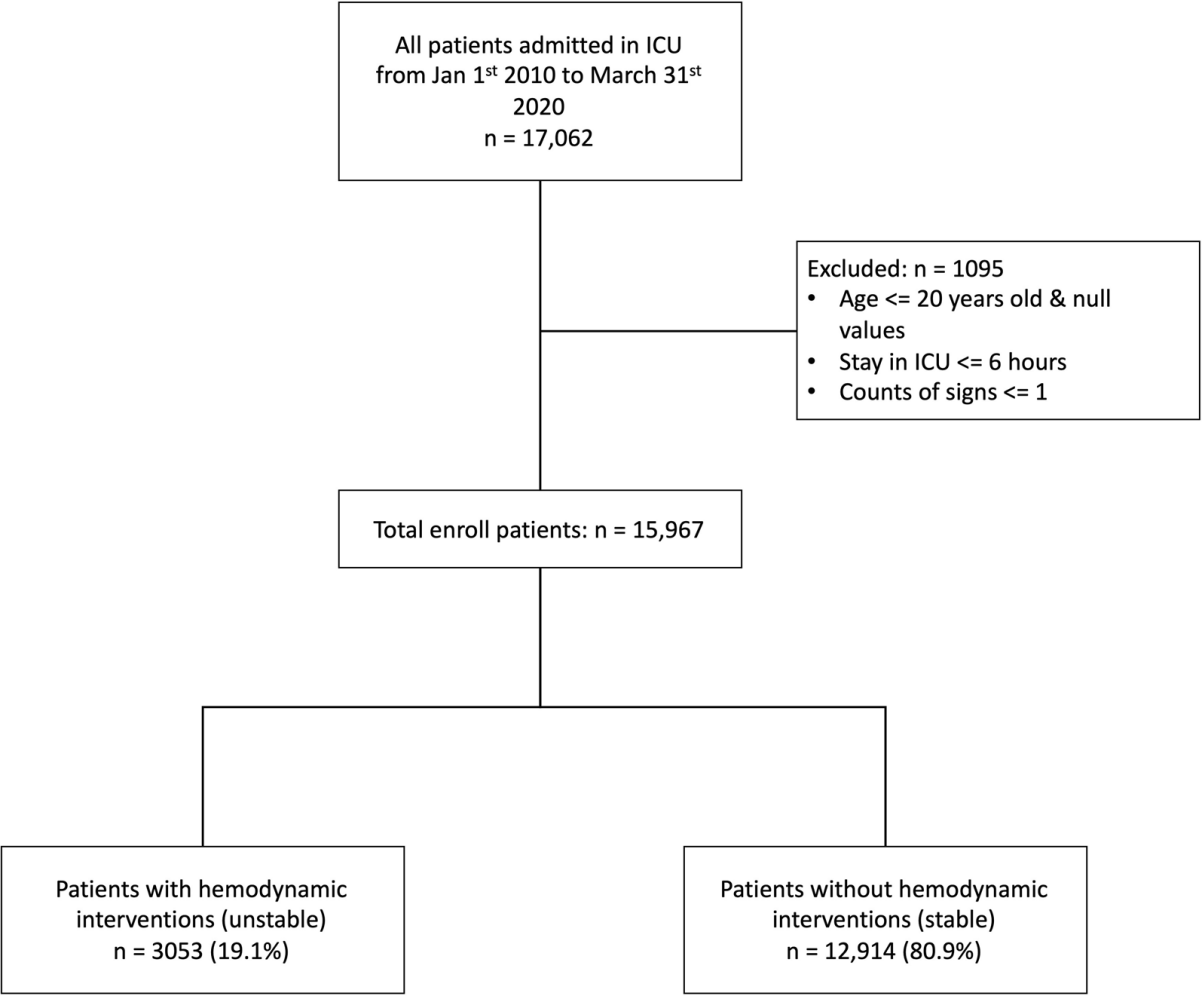
Flow diagram for inclusion and exclusion criteria of patients

This retrospective study included mixed-ICU patients age > 20 years admitted to Taipei Veteran General Hospital (TPEVGH) between January 1, 2010, and March 31, 2020 (TPEVGH cohort for short). We excluded the patients who had incomplete data profiles or stayed in ICU for less than 6 h aligned with HSI development cohort (US cohort for short) (Fig. 1). We extracted those patients’ data from ICU clinical information system, i.e., Philips IntelliSpace Critical Care and Anesthesia (ICCA). This study was reviewed and approved by both ethical committee of TPEVGH (No. 2020-09-001AC) and Philips Internal Committee of Biomedical Experiments (ICBE-2-36635).

### Definition of hemodynamic instability and annotation rules

Hemodynamic instability was labeled as any administration of inotropes, vasopressor, significant fluid support, and/or blood transfusions, which were aligned with US cohort on categories. Differences in annotating details to follow TPEVGH practices are shown in Table 1. A patient with hemodynamic instability can have multiple unstable segments annotated by hemodynamic interventions during ICU stay (Additional file 1: Fig. S1), and only first segment after 6 h in ICU was used for this validation. We also excluded hemodynamic instability segments administrated within first 6 h in ICU into account.

**Table 1:** Criteria to annotate hemodynamic instability and differences between US and TPEVGH cohort

Hemodynamic instability was annotated by hemodynamic interventions under any of the following criteria	
US Cohort*	TPEVGH Cohort**
Administration of any quantity of any of the following inotropic and vasopressor medications:	Administration of any quantity of any of the following inotropic and vasopressor medications:
1. Dobutamine	1. Dobutamine
2. Dopamine	2. Dopamine
3. Epinephrine	3. Epinephrine
4. Levophed	4. Levophed
5. Neosynephrine	5. Norepinephrine
6. Norepinephrine	6. Phenylephrine
7. Phenylephrine	7. Vasopressin
8. Vasopressin	
Administration of fluid therapy (colloid or crystalloid) in the following dosages:	Administration of fluid therapy (colloid or crystalloid) in the following dosages:
1. 2400 cc in 8 h	1. The same as US
2. 3000 cc in 12 h	2. 25% Albumin 200 cc with 2 h
Administration of packed red blood cells (PRBCs) in either of the following dosages:	Administration of packed red blood cells (PRBCs) in either of the following dosages:
1. 800 cc PRBC over course of 24 h	1. PRBC > 1500 cc with 24 h
2. 500 cc in 2 hours followed by fluid therapy within 12 h. (What qualifies as “fluid therapy" is described in this table, titled "Administration of Fluid Therapy.")	2. PRBC 500 cc + FPP 500 cc + PLT Pheresis 500 cc within 6 h

*US cohort which was used to developed Hemodynamic Stability Index (HSI)

**TPEVGH Cohort, Taipei Veteran General Hospital Cohort for this HSI external validation study

### HSI model

The HSI model is an early detection model to predict hemodynamic instability, which was developed by Philips Research North America based on patients’ data from eICU Research Institute (eRI) dataset [9, 14]. This model was developed with an ensemble of interpretable decision trees to obtain a single real-time risk score to continuously monitor the hemodynamic status with 33 routinely measured physiological variables. The profiles of 33 features including vital signs, laboratory results, blood gas measurements, and ventilation settings are presented in Additional file 1: Table S1 . HSI demonstrated generalizability across clinically relevant patient populations on a retrospective validation set, and better accuracy in predicting hemodynamic interventions 1 h in advance compared to single parameters like SBP and shock index (AUROC was 0.82 compared to 0.69 and 0.39 for shock index and SBP) [9]. HSI provided a risk score even with a subset of missing variables and was calculated on an hourly interval to detect unstable segments.

### Data processing

All variables passed through a plausibility filter (Additional file 1: Table S1) to check whether their values were in the physiologically valid range and outliers were replaced as missing. For the patient in unstable group, time-varying data of each variable were extracted every hour that preceded the onset time of hemodynamic interventions. For the patient in stable group, data of each variable were extracted within 5-h observation window after patients’ admission to ICU. The missing values, which were not available at that time point of extraction, were processed following the method reported in HSI model study [9, 15] (Additional file 1: Table S2 ). The noninvasive blood pressures were used to impute as the invasive variables when invasive measurements were not available. The fraction of inspired oxygen (FiO_2_) was imputed to room oxygen level of 0.21, and the rest was kept missing since the HSI model allowed that some measurements were not available.

### Statistics and case-mix effect

Patients’ characteristics and baseline characteristics including demographics, admission type of ICU, ICU unit type, APACHEII score and admission sources were compared between unstable group with hemodynamic interventions and stable group without hemodynamic interventions. Nonparametric tests were applied since the population data did not have a normal distribution [16]. Kruskal–Wallis test was used to test the significance of continuous variables in the form of median and quartiles across both groups. Fisher's exact test was used to test the categorical variables. The model performance was assessed using the area under the receiver operating characteristic curve (AUROC), and Delong method was used to calculate 95% confidence interval (CI) of AUROC and to compare with single indicators like SBP and shock index [17]. The hemodynamic instability cutoff was set by selecting optimal threshold with high recall, i.e., sensitivity, acceptable specificity, and lead time before intervention was calculated to indicate when patients were firstly identified as high risk of hemodynamic instability. In addition, calibration plot was visualized to assess the agreement between predictions and observations. Additionally, to assess case-mix effect, i.e., the effect of the difference in predictor values’ distribution on predictive performance between the development and validation cohort [18], we calculated the median for each continuous variable, respectively, and compared with ones in US cohort.

## Results

Of 17,062 ICU stays, 15,967 patients who admitted to TPEVGH ICU were identified in our retrospective study over 10 years. In total, 3053 (19.1%) patients were in the unstable group, and the rest 80.9% (12,914) patients were in the stable group (Fig. 1).

**Table 2:** Patient characteristics comparison between unstable and stable group

Characteristics	Unstable	Stable	Overall	*p* value
	*N* = 3053	*N* = 12,914	*N* = 15,967	
Age, median [*Q*1, *Q*3]	70 [58, 82]	70 [56, 82]	70 [57, 82]	0.237^a^
Gender *n* (%)				
Female	1058 (34.6)	4524 (35.0)	5582 (35.0)	0.71^b^
Male	1995 (65.4)	8390 (65.0)	10,385 (65.0)	
APACHEII, median [*Q*1, *Q*3]	25 [20, 31]	21 [15, 26]	22 [16, 28]	< 0.001^a^
Length of stay (days), median [*Q*1, *Q*3]	12 (7, 19)	5 (2, 8)	6 (3, 10)	< 0.001^a^
Admission type *n* (%)				
Emergency	1228 (40.2)	5735 (44.4)	6963 (43.6)	0.012^b^
Not emergency	1427 (46.7)	5965 (46.2)	7392 (46.3)	
Other	398 (13.1)	1207 (9.4)	1605 (10.1)	
ICU *n* (%)				
Surgical	1007 (33.0)	5277 (40.9)	6284 (39.4)	< 0.001^b^
Medical	2046 (67.0)	7637 (59.1)	9683 (60.6)	
ICU mortality *n* (%)				
Survivors	1805 (59.1)	11,533 (89.3)	13,338 (83.5)	< 0.001^b^
Death	1248 (40.9)	1381 (10.7)	2629 (16.5)	
Admission source, *n* (%)				
Cardiovascular medical	50 (1.6)	1219 (9.4)	1269 (7.9)	< 0.001^b^
Cardiovascular surgical	37 (1.2)	269 (2.1)	306 (1.9)	
Gastrointestinal medical	373 (12.2)	1331 (10.3)	1704 (10.7)	
Gastrointestinal surgical	536 (17.6)	2602 (20.1)	3138 (19.7)	
Metabolic/endocrinology medical	196 (6.4)	606 (4.7)	802 (5.0)	
Neurologic medical	5 (0.2)	20 (0.2)	25 (0.2)	
Neurologic surgical	3 (0.1)	24 (0.2)	27 (0.2)	
Others medical	1419 (46.5)	4448 (34.4)	5867 (36.7)	
Others surgical	329 (10.8)	1865 (14.4)	2194 (13.7)	
Respiratory medical	3 (0.1)	13 (0.1)	16 (0.1)	
Respiratory surgical	11 (0.4)	23 (0.2)	34 (0.2)	
Trauma surgical	91 (3.0)	494 (3.8)	585 (3.7)	

^a^Kruskal–Wallis test

^b^Fisher's exact test

Table 2 shows that patients in stable group had significantly higher APACHEII score in first 24 h admitted to ICU (*p* < 0.001), higher mortality (*p* < 0.001), and longer ICU stay (*p* < 0.001). Compared to stable patients, unstable patients admitted less from emergency (*p* < 0.001), and more from medical ICU (*p* < 0.001). However, no significant differences were found in age (*p* = 0.237) and gender (*p* = 0.71) between two groups. Additionally, patients with hemodynamic interventions have significantly lower blood pressure, hemoglobin, and hematocrit (*p* < 0.001), higher heart rate, central venous pressure (CVP) (*p* < 0.001), blood urine nitrogen (BUN), lactate, aspartate transaminase (AST), creatinine, peak airway pressure, mean airway pressure, and FiO_2_ (*p* < 0.001). The detailed baseline data of 33 features between two groups were calculated within 24 h after ICU admission and are shown in Additional file 1: Table S3.

### Model validation and performance

**Fig. 2:**
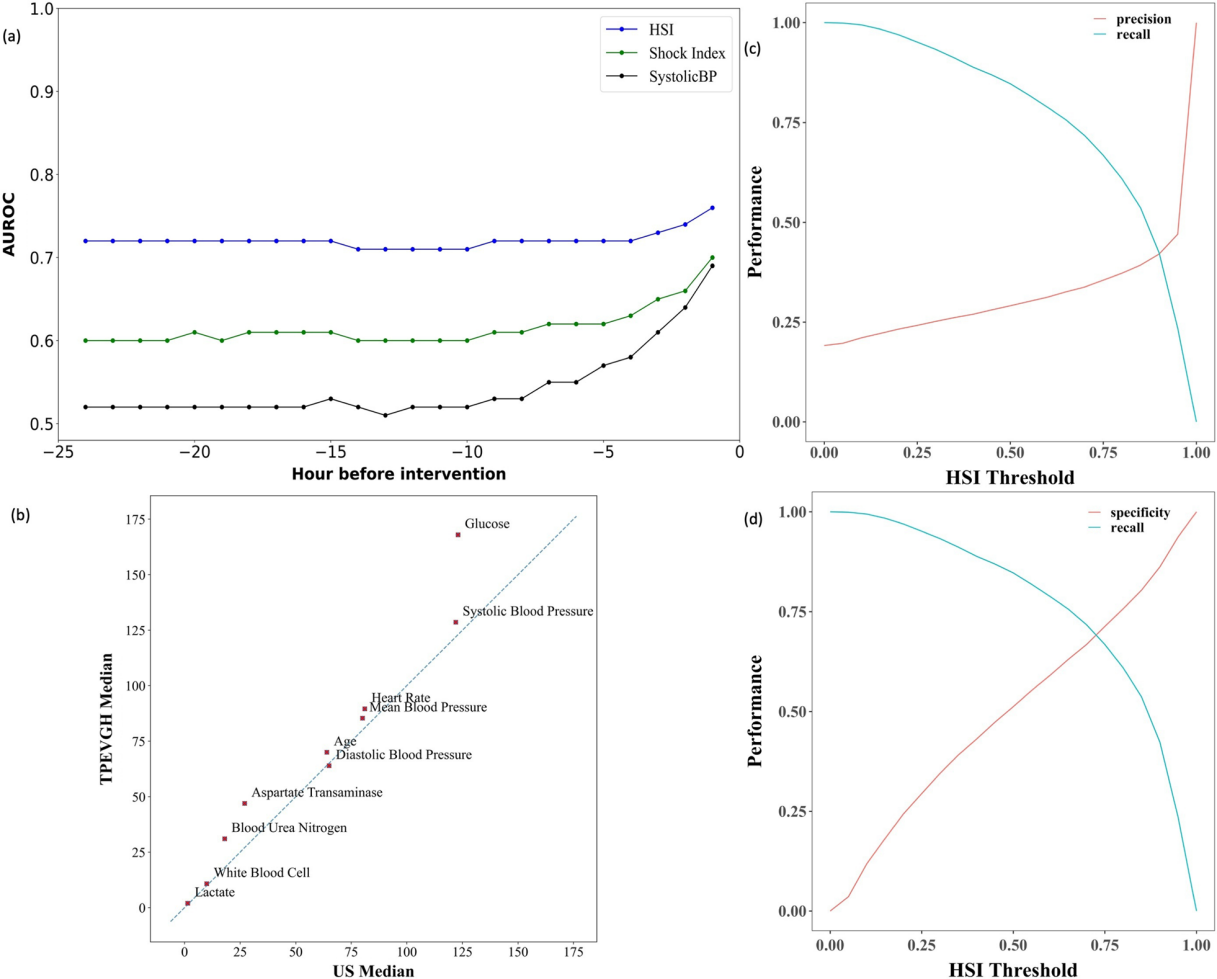
Model and threshold performance plots. **a** Time-varying results of HSI model at different prediction times before hemodynamic interventions, compared to Shock Index and systolic blood pressure (systolic BP); **b** median comparison between the US cohort and TPEVGH cohort (not all the features are listed due to complications of whole figure); **c** HSI recall–precision curve of TPEVGH cohort; **d** HSI recall–specificity curve of TPEVGH cohort

Hemodynamic instability segments were annotated by interventions including inotropic/vasopressor medication, fluid therapy, and/or blood transfusion. AUROC of HSI on TPEVGH cohort 1 hour before the intervention was 0.76 (95% CI 0.75–0.77) according to annotation rules with hemodynamic interventions (19.1%), which performed better than Shock index (AUROC 0.7; 95% CI 0.69–0.71) and SBP (AUROC 0.69; 95% CI 0.68–0.70). Details are shown in Additional file 1: Table S4. And we also found out that only 92 patients with hemodynamic interventions in TPEVGH cohort did not administrate vasopressor/inotropic medications and the AUROC remained the same by excluding these 92 patients from unstable group. HSI score was hourly calculated by using 33 features in 24 h before first hemodynamic intervention. Time-varying results of HSI score show that it outperformed shock index and SBP even up to 24 h before first hemodynamic interventions (Fig. 2a).

### Sensitivity analysis and selection of optimal threshold

The output of HSI model is the probability to indicate a risk of hemodynamic interventions. The higher probability is the higher risk of hemodynamic instability (unstable). We selected the alarm threshold of HSI score based on the performance of HSI on TPEVGH cohort. The threshold is used as a cutoff of HSI score to get unstable segments. Figure 2c shows the recall–precision curve of HSI model. Ideally, the intersection point is the break-even point to get an optimal threshold without compromising the precision, but this point was not the best case since recall (sensitivity of hemodynamic instability) was lower than 50%. From the confusion matrix shown in Additional file 1: Table S5 , the threshold was in the range of 0.65 to 0.70 when the drop of recall and increase in specificity could be balanced. To enhance the recall of prediction model as an early warning alarm, we finally chose 0.7 as the threshold for TPEVGH cohort. And it is also in the range of break-even point between specificity and recall in Fig. 2d. The calibration plot also indicated that HSI had better agreement between predictive and observational hemodynamic instability risk when the threshold was over 0.7, than ones under 0.7, especially ones over 0.82 (shown in Additional file 1: Fig. S2).

### Evaluation of potential clinical benefit and lead time before intervention

**Fig. 3:**
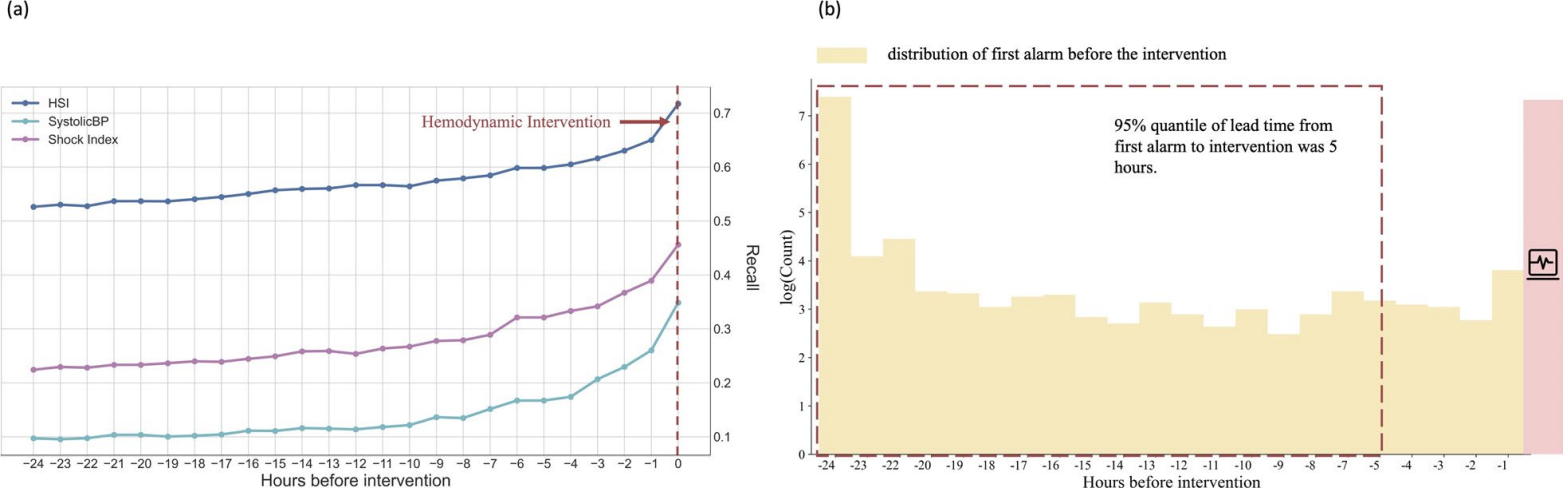
Time-varying true alarms and leading time plots. **a** The fraction of events that correctly trigger an alarm is reported per hour in 24 h before any hemodynamic intervention occurs. **b** The distribution of timing of the first alarm in the 24 h before an event. 95% unstable patients can be identified over 5 h in advance to interventions

We have selected 0.7 as a threshold and calculated HSI scores to predict hemodynamic interventions hour by hour. Of 15,967 ICU stays, 3053 patients administrated interventions and 2190 patients (72%) can be identified by using HSI model 1 h before the interventions. The fraction of true alarm decreased to below 60% when it is over 6 h in advance to hemodynamic interventions (Fig. 3a). HSI has the higher correctly trigger rates than other two single parameters including shock index and SBP. The false alarm rate kept around 0.3 through all 24 h before interventions (Additional file 1: Fig. S3). Once alert was addressed at first time of HSI score > 0.7, 95% unstable patients can be identified over 5 h in advance to interventions (Fig. 3b).

### Model performance in different subgroups of patients

**Fig. 4:**
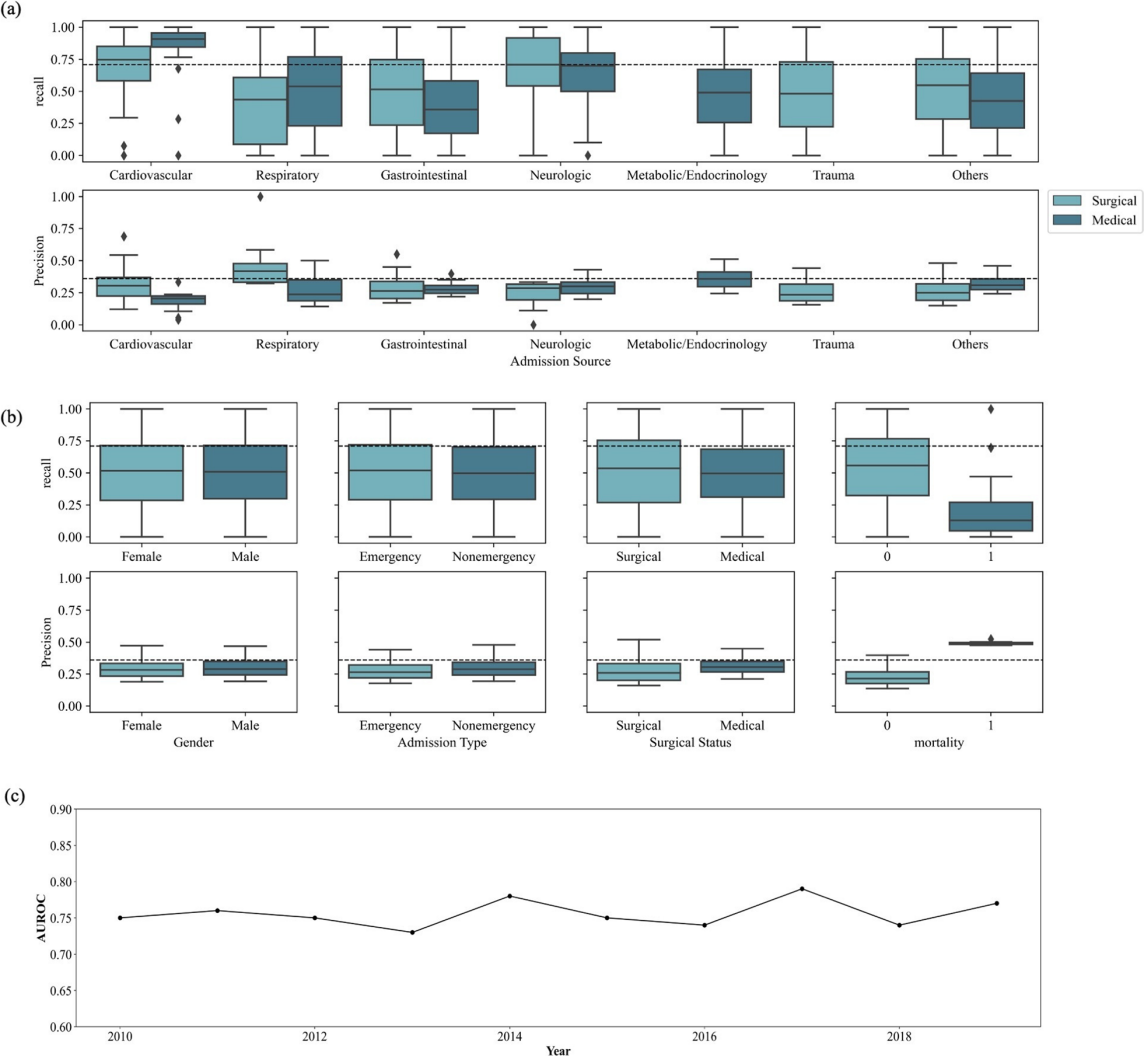
Model performance in different subgroup cohorts. **a** Model performance in different admission source subgroups of TPEVGH cohort. * means outliers, identified by 1.5*IQR; **b** model performance in different subgroups of TPEVGH cohort on gender, admission type, surgical status, and death. * means outliers, identified by 1.5*IQR; **c** HSI model AUROC performance by year of TPEVGH cohort

We evaluated the HSI model in subgroups with different admission sources and compared the difference between medical and surgical ICU as well in Fig. 4a. Patients admitted from cardiology department got the better results with AUROC of 0.89 (95% CI: 0.87–0.92), and highest recall in surgical group admitted from cardiology department. AUROC values in other admission source subgroups were close to 0.76 (0.72–0.78), which was the comparable performance with the whole cohort (Additional file 1: Table S6). Figure 4a also indicates small variance of precision across different admission sources. Figure 4b shows that the lower recall and higher precision were found in death group.

### Model performance over time

The performance of HSI over time was also plotted year by year as shown in Fig. 4c. The AUROC range was around 0.70 to 0.80. There was the higher AUROC value in 2017 and the worst AUROC value in 2013. Since there are only 3 months in 2020, we combined them in 2019 data to calculate AUROC.

### Inspection of dataset shift on case-mix effect

Dataset shift in terms of the difference in the median of predictors was observed in individual features from HSI development cohort in US to our external validation cohort in TPEVGH. The notable feature median differences were blood glucose with 37%, AST with 74%, and BUN with 72% higher in TPEVGH cohort. The rest features had similar distributions in median as shown in Fig. 2b, and detailed distribution shift between HSI development cohort in US and our external validation cohort in TPEVGH is also shown in Additional file 1: Table S7.

## Discussion

The HSI model provides an early warning of hemodynamic instability by detecting hemodynamic interventions. The external validation of this model outperforms traditional measures like shock index and SBP (AUROC: 0.76 vs. 0.70 vs. 0.69, respectively). It is still in an acceptable range and is worth to be implemented in clinical setting, though the AUROC decreased from 0.82 in US cohort to 0.76 in TPEVGH cohort. The higher probability predicted by HSI model indicates the larger risk of hemodynamic instability. The threshold of 0.7 was selected as cutoff based on the performance on TPEVGH cohort with higher recall (0.72) without compromising a lot in the drop of specificity (0.67). Calibration plots showed that the overall performance of HSI model underestimated the risk of hemodynamic instability in TPEVGH, whereas the patients who were predicted as hemodynamic instability (probability > 0.7) had better agreement between predictive and observation values. The model tends to underestimate the risk when training cohort is in a lower incidence; however, the incidence was comparable between training and external validation cohort (19% vs. 18%) [19]. Heterogeneity of predictors’ effect and difference in case mix can be the reason of underestimation. We still have space to optimize the performance of HSI by retraining. Time-varying results of HSI model showed the promising future for clinical applications since 95% patients with hemodynamic instability could be detected over 5 h before hemodynamic interventions with the false alarm rate being remained at 30%. Besides, we also found that even some common measured features were still missing in some features such as 22.1% missing of lactate in TPEVGH cohort, although dramatically less than US cohort (Additional file 1: Table S2). In contrast to prior work by Hyland et al., the full model with 112 variables leads to high ratio of imputation for missing features and the imputation results in overrating patients’ risk and increasing high ratio of false alert [8].

This study is the first work on hemodynamic instability to externally validated in Asian cohort by the independent researchers. Although features and model aligned with original HSI development study, annotation criteria with minor adjustment, performance reduction was observed. Annotation criteria were adjusted to follow the clinical practices of TPEVGH. In this case, we can know if HSI model can be applicable in TPEVGH with their practices on hemodynamic interventions. And we also found out that 92 patients in TPEVGH cohort administrated only fluid therapy and/or blood transfusion, and the performance of HSI was not affected once we excluded these 92 patients. Prediction models frequently performed worse in external cohort than in development cohort, due to the difference in outcome incidence, heterogeneity of predictors’ effect, and difference in the distribution of predictors’ value [12, 20, 21]. In our study, the incidence of hemodynamic instability is not largely deviated from original US cohort (19% vs. 18%), and performance was not changed after calibrating the incidence. Further, distribution of some features in terms of median shifts a lot. The largest differences in median were blood glucose with 37% increase, AST with 74% increase, and BUN with 72% increase (Fig. 2b).

According to the current critical care glucose control guidelines, the glucose level of critically illness patient should be controlled within 150–180 mg/dl [22]. However, due to severe complications of hypoglycemia, glucose control is not so restricted in TPEVGH. Median sugar level in US cohort is about 123 mg/dl and that in TPEVGH cohort is about 168 mg/dl. Both are within acceptable range. However, it could influence the performance owing to case-mix effect. We also noticed that BUN level is also relatively higher in TPEVGH population 31 mg/dl than in US cohort 18 mg/dl which may also contribute to the impairment of model's performance. We reviewed the previous studies showing that chronic kidney disease (CKD) and impaired renal function were closely related to higher mortality rates in ICU patients [23]. The prevalence of total CKD was 15.5% in Taiwan which was higher than the rest of the world [24]. As to AST, the frequency of acquired liver injury and failure in critical illness has been significantly increased. Liver injury and failure are observed in up to 20% of patients in ICU [2]. The median AST level in US cohort is about 27U/L and that in TPEVGH cohort is about 47U/L. In TPEVGH, the normal range was set as below 40U/L. According to the review of Thomas Horvatits et al. [25], we believe that the higher AST level in critical illness patients is reasonable. In contrast, the median AST of US cohort is in the middle of normal range which seems to be unexplainable. Different laboratory and test machines might have different results and reference ranges, which may bring to a different conclusion. We also reviewed the incidence of hepatitis in critical illness. There is no available data of in-ICU-hepatitis incidence so far. According to a previous study, the incidence of sepsis-associated liver injury is 34.7% which is not rare in ICU [26].

We also performed the analysis according to different admission source (Fig. 4a). As compared to the US cohort, this external validation by TPEVGH cohort still shows great recall on cardiovascular group either in medical or in surgical patients. When it comes to neurological group, TPEVGH cohort remains in a higher recall up to 70%; however, it is lower in US cohort (< 70%). The explanation may be due to differences in admission characteristics of patients. In US cohort, the neurological patients received vasoactive agent is not necessarily due to hemodynamic instability. In TPEVGH, typical patients from neurological department will not be transferred in the ICU, and the baseline of noninvasive SBP was 112.3 mmHg in median (*Q*1: 101.8, *Q*3: 127.2). Most of the patients were admitted into our ICU due to sepsis or medical problems.

We also investigated subgroup analysis of gender, admission type, surgical status, and mortality. No differences of performance on recall and precision were identified in gender, admission type, and surgical status, which indicated the robust performance of HSI model in subgroups. Low recall and high precision were found in death group which were owing to model characteristics. The performance of HSI over year was also stable to the average and demonstrated robustness of HSI over time.

## Limitations

The key limitation of this study is that we performed on cohort from a single medical center, although the cohort was large and over 10 years. Another limitation is the constraints of HSI model itself. Hemodynamic variables like cardiac output, stroke volume, and stroke volume variation would likely add predictive power to HSI once integrated with clinical information system [27, 28]. Other features such as sonography, medical images, and even drugs such as antibiotics may also play an important part of prediction.

Our next step is to optimize the HSI model to overcome the underestimated status and perform federated learning between hospitals to gain a generalized result. We also will integrate the model into our clinical information system to continuously collect clinical data, validate, and further optimize our model.

## Conclusion

This external validation indicates that the HSI has acceptable discrimination but underestimates the risk of stable patients in predicting the onset of hemodynamic instability. The leading time of 5 h could be used as a clinical alarm. The optimized AI model will be further validated to address the case-mix effect by dataset shift.

## Supplementary Information

**Additional file 1.**.** Fig. S1**. Annotation rules for hemodynamic intervention.** Table S1**. Clinical variables used in HSI and plausibility filter for each variable.** Table S2**. Missing rate of clinical variables.** Table S3**. Baseline characteristics comparison between unstable and stable patients.** Table S4**. Performances of HSI model, shock index and systolic blood pressure in TPEVGH cohort.** Table S5**. Confusion matrix of HSI model under different threshold.** Table S6**. Subgroup performance of HSI model in TPEVGH.** Table S7**. Median comparison between the US cohort and TPEVGH cohort.

## Data Availability

The datasets used and/or analyzed during the current study are available from the corresponding author on reasonable request.

## Abbreviations

HSI: Hemodynamic Stability Index
TPEVGH: Taipei Veteran General Hospital
AUROC: Area under the receiver operating characteristic curve
SBP: Systolic blood pressure
ICU: Intensive care unit
CI: Confidence interval
US: United States
MIMIC III: Medical information mart for intensive care III
ICCA: Philips IntelliSpace Critical Care and Anesthesia
ICBE: Philips Internal Committee of Biomedical Experiments
eRI: eICU Research Institute
FiO_2_: Fraction of inspiration oxygen
CVP: Central venous pressure
BUN: Blood urine nitrogen
AST: Aspartate transaminase
CKD: Chronic kidney disease
BDC: Big data center
