# Correction: External validation of a machine learning model to predict hemodynamic instability in intensive care unit

**DOI:** 10.1186/s13054-026-05980-4

**Published:** 2026-09-29

**Authors:** Chiang Dung‑Hung, Tian Cong, Jiang Zeyu, Ou‑Yang Yu‑Shan, Lin Yung‑Yang

**Affiliations:** 1 Department of Critical Care Medicine, Taipei Veteran General Hospital, No. 201, Section 2, Shih‑Pai Road, Taipei, 11217 Taiwan; 2 School of Medicine, National Yang Ming Chiao Tung University, Taipei, Taiwan; 3 Philips Research China, Shanghai, 200072 China

**Correction: Critical Care (2022) 26:215**

**https://doi.org/10.1186/s13054-022-04088-9**

Following publication of the original article [1], the authors identified an error in the author name of Lin Yung‑Yang.

The incorrect author name is: Lin Yung‑Yan

The correct author name is: Lin Yung‑Yang

The author group has been updated above and the original article [1] has been corrected.
