# Supplementary material for: External validation of a machine learning model to predict hemodynamic instability in intensive care unit
Source: Crit Care. 2022 Jul 14;26:215. doi: 10.1186/s13054-022-04088-9 (PMC9281065; doi:10.1186/s13054-022-04088-9)
Supplement: Supplementary file 1 — Additional file 1.. Fig. S1. Annotation rules for hemodynamic intervention. Table S1. Clinical variables used in HSI and plausibility filter for each variable. Table S2. Missing rate of clinical variables. Table S3. Baseline characteristics comparison between unstable and stable patients. Table S4. Performances of HSI model, shock index and systolic blood pressure in TPEVGH cohort. Table S5. Confusion matrix of HSI model under different threshold. Table S6. Subgroup performance of HSI model in TPEVGH. Table S7. Median comparison between the US cohort and TPEVGH cohort. [file 13054_2022_4088_MOESM1_ESM.docx]

**Fig. S1. Annotation rules for hemodynamic intervention**


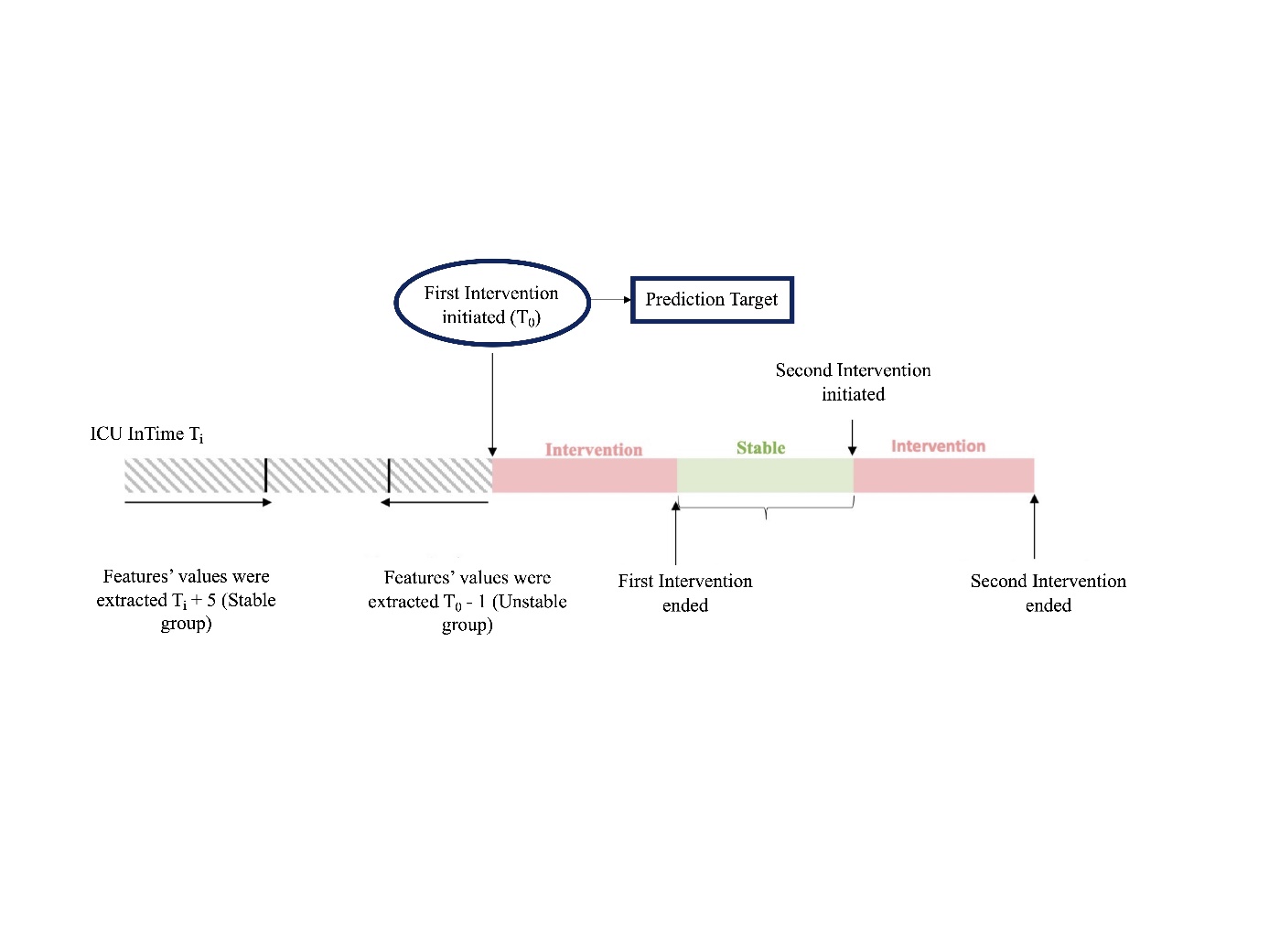


**Table S1. Clinical variables used in HSI and plausibility filter for each variable**

| **Characteristics** | **Unit** | **Plausibility Low** | | **Plausibility High** |
| --- | --- | --- | --- | --- |
| Age | years | | 20 | ~ |
| **Vital Signs** | | | | |
| Invasive diastolic blood pressure | mmHg | | 0 | 250 |
| Invasive systolic blood pressure | mmHg | | 0 | 250 |
| Invasive mean blood pressure | mmHg | | 0 | 250 |
| Non-invasive diastolic blood pressure | mmHg | | 0 | 250 |
| Non-invasive systolic blood pressure | mmHg | | 0 | 250 |
| Non-invasive mean blood pressure | mmHg | | 0 | 250 |
| Heart Rate | bpm | | 0 | 200 |
| Central venous pressure | mmHg | | 0 | 50 |
| Temperature | degrees (℃) | | 30 | 45 |
| Shock Index | bpm/mmHg | | 0 | 5 |
| **Lab and Blood Gas Measurement** | | | | |
| Creatinine | mg/dL | | 0 | 20 |
| Blood Urea Nitrogen | mg/dL | | 0 | 500 |
| Total Bilirubin | mg/dL | | 0 | 50 |
| Potassium | mmol/L | | 2 | 10 |
| Sodium | mmol/L | | 100 | 200 |
| Calcium | mg/dL | | 0 | 50 |
| SaO_2_ | % | | 0 | 100 |
| PaCO_2_ | mmHg | | 0 | 200 |
| CO_2_ | mmol/L | | 0 | 50 |
| Base Excess | mEq/L | | -30 | 30 |
| Hemoglobin | g/dL | | 2 | 22 |
| Hematocrit | % | | 10 | 80 |
| Magnesium | mg/dL | | 0 | 20 |
| Partial thromboplastin time | seconds | | 0 | 250 |
| Aspartate transaminase | Units/L | | 0 | 30000 |
| Ionized Calcium | mmol/L | | 0 | 3 |
| Bands | % | | 0 | 100 |
| Glucose | mg/dL | | 0 | 1000 |
| Lactate | mg/dL | | 0 | 500 |
| WBC | u/uL | | 0 | 60000 |
| Eosinophils | % | | 0 | 100 |
| Basophils | % | | 0 | 20 |
| **Ventilation Settings** | | | | |
| Peak Airway Pressure | mmHg | | 0 | 100 |
| Mean Airway Pressure | mmHg | | 0 | 90 |
| FiO_2_ | % | | 21 | 100 |

**Table S2. Missing rate of clinical variables**

| **Characteristics** | **TPEVGH Cohort Missing rate (%)** | **United States Cohort Missing rate (%)** |
| --- | --- | --- |
|  |  |  |
| Age | 0 | 2 |
| **Vital Signs** | | |
| Invasive diastolic blood pressure | 11 | 77 |
| Invasive systolic blood pressure | 11 | 77 |
| Invasive mean blood pressure | 10 | 78 |
| Non-invasive diastolic blood pressure | 0 | 1 |
| Non-invasive systolic blood pressure | 0 | 1 |
| Non-invasive mean blood pressure | 0 | 1 |
| Heart rate | 0 | 0 |
| Central venous pressure | 41 | 86 |
| Temperature | 1 | 79 |
| Creatinine | 15 | 3 |
| Blood urine nitrogen | 17 | 3 |
| Total Bilirubin | 37 | 27 |
| Potassium | 6 | 3 |
| Sodium | 6 | 3 |
| Calcium | 64 | 4 |
| SaO_2_ | 12 | 57 |
| PaCO_2_ | 12 | 55 |
| CO_2_ | 12 | 4 |
| Base Excess | 14 | 71 |
| Hemoglobin | 7 | 3 |
| Hematocrit | 59 | 3 |
| Magnesium | 53 | 43 |
| Partial thromboplastin time | 61 | 36 |
| Aspartate transaminase | 51 | 28 |
| Ionized Calcium | 45 | 81 |
| Bands | 15 | 91 |
| Glucose | 24 | 3 |
| Lactate | 22 | 83 |
| WBC | 13 | 4 |
| Eosinophil (%) | 16 | 60 |
| Basophil (%) | 17 | 60 |
| **Ventilation Settings** | | |
| Peak Airway Pressure | 31 | 90 |
| Mean Airway Pressure | 32 | 92 |
| FiO_2_ | 24 | 70 |

* The same method with United States bench-mark model for dealing with missing values is used to this analysis, if no available values at that time point were extracted, missing values would be kept except that invasive blood pressures were imputed by non-invasive blood pressure values and fraction of inspiration oxygen (FiO_2_) was filled by 21% when missing.

**Table S3. Baseline characteristics comparison between unstable and stable patients**

| **Characteristics** | **Overall** |  | **Stable** |  | **Unstable** |  | **P-Value** |
| --- | --- | --- | --- | --- | --- | --- | --- |
|  | **N =15967** |  | **N = 12914** |  | **N= 3053** |  |  |
| Age, median [Q1,Q3] | 70.0 [57.0,82.0] |  | 70.0 [56.0,82.0] |  | 70.0 [58.0,82.0] |  | 0.237^a^ |
| **Vital Signs** | | | | | | | |
| Invasive diastolic blood pressure, median [Q1,Q3] | 60.6 [53.7,69.1] |  | 61.2 [54.1,69.7] |  | 58.6 [52.3,66.2] |  | <0.001^a^ |
| Invasive systolic blood pressure, median [Q1,Q3] | 118.9 [105.3,137.0] |  | 120.4 [106.2,138.6] |  | 113.9 [103.0,130.1] |  | <0.001^a^ |
| Invasive mean blood pressure, median [Q1,Q3] | 79.6 [71.1,90.2] |  | 80.5 [71.7,91.3] |  | 76.3 [69.1,85.9] |  | <0.001^a^ |
| Non-invasive diastolic blood pressure, median [Q1,Q3] | 62.3 [55.2,70.3] |  | 63.0 [55.6,70.9] |  | 60.0 [53.5,67.4] |  | <0.001^a^ |
| Non-invasive systolic blood pressure, median [Q1,Q3] | 112.3 [101.8,127.2] |  | 113.5 [102.4,128.7] |  | 107.9 [99.6,120.1] |  | <0.001^a^ |
| Non-invasive mean blood pressure, median [Q1,Q3] | 75.8 [68.5,84.7] |  | 76.5 [68.9,85.5] |  | 72.8 [66.9,81.1] |  | <0.001^a^ |
| Heart rate, median [Q1,Q3] | 91.6 [79.0,105.2] |  | 90.0 [78.0,103.5] |  | 98.0 [84.3,111.1] |  | <0.001^a^ |
| CVP, median [Q1,Q3] | 9.8 [7.5,12.4] |  | 9.5 [7.3,12.2] |  | 10.4 [8.3,13.2] |  | <0.001^a^ |
| Temperature, median [Q1,Q3] | 37.0 [36.5,37.4] |  | 37.0 [36.5,37.4] |  | 37.0 [36.5,37.5] |  | <0.001^a^ |
| Shock Index, median [Q1,Q3] | 0.8 [0.6,1.0] |  | 0.7 [0.6,0.9] |  | 0.9 [0.7,1.1] |  | <0.001^a^ |
| **Lab and Blood Gas Measurement** | | | | | | | |
| Creatinine, median [Q1,Q3] | 1.5 [1.0,2.8] |  | 1.4 [0.9,2.5] |  | 2.0 [1.2,3.8] |  | <0.001^a^ |
| Blood urine nitrogen, median [Q1,Q3] | 30.0 [17.0,54.0] |  | 28.0 [16.0,49.0] |  | 42.0 [25.0,71.0] |  | <0.001^a^ |
| T-Bilirubin, median [Q1,Q3] | 0.9 [0.5,2.0] |  | 0.9 [0.5,1.8] |  | 1.1 [0.6,2.8] |  | <0.001^a^ |
| K+, median [Q1,Q3] | 3.8 [3.4,4.3] |  | 3.8 [3.4,4.2] |  | 3.8 [3.3,4.4] |  | 0.046^a^ |
| Na+, median [Q1,Q3] | 139.0 [135.7,142.7] |  | 139.0 [136.0,142.3] |  | 139.0 [135.0,143.0] |  | 0.215^a^ |
| Ca, median [Q1,Q3] | 7.8 [7.2,8.4] |  | 7.8 [7.2,8.4] |  | 7.8 [7.1,8.5] |  | 0.33^a^ |
| SaO_2_, median [Q1,Q3] | 98.7 [97.2,99.5] |  | 98.8 [97.4,99.5] |  | 98.4 [96.8,99.4] |  | <0.001^a^ |
| PaCO_2_, median [Q1,Q3] | 29.6 [26.3,33.5] |  | 29.6 [26.4,33.5] |  | 29.3 [26.0,34.0] |  | 0.266^a^ |
| CO_2_, median [Q1,Q3] | 20.4 [18.0,23.0] |  | 20.5 [18.1,23.0] |  | 20.1 [17.6,23.0] |  | 0.001^a^ |
| Base Excess, median [Q1,Q3] | -2.6 [-5.2,0.2] |  | -2.5 [-5.1,0.2] |  | -2.9 [-5.6,0.0] |  | <0.001^a^ |
| Hemoglobin, median [Q1,Q3] | 9.4 [8.2,11.1] |  | 9.6 [8.3,11.2] |  | 8.9 [7.9,10.3] |  | <0.001^a^ |
| Hematocrit, median [Q1,Q3] | 28.0 [24.3,33.0] |  | 28.6 [24.7,33.4] |  | 26.5 [23.0,30.6] |  | <0.001^a^ |
| Mg++, median [Q1,Q3] | 2.0 [1.7,2.2] |  | 1.9 [1.7,2.2] |  | 2.0 [1.8,2.3] |  | <0.001^a^ |
| APTT, median [Q1,Q3] | 34.2 [29.6,42.5] |  | 33.6 [29.2,41.8] |  | 35.8 [31.1,44.7] |  | <0.001^a^ |
| AST, median [Q1,Q3] | 48.0 [25.0,134.1] |  | 45.0 [24.0,127.0] |  | 57.2 [29.0,157.0] |  | <0.001^a^ |
| iCa, median [Q1,Q3] | 4.5 [4.2,4.7] |  | 4.5 [4.2,4.7] |  | 4.5 [4.2,4.7] |  | 0.624^a^ |
| Band (%), median [Q1,Q3] | 0.0 [0.0,0.0] |  | 0.0 [0.0,0.0] |  | 0.0 [0.0,0.0] |  | <0.001^a^ |
| Glucose, median [Q1,Q3] | 162.5 [125.0,217.4] |  | 162.0 [125.0,216.7] |  | 163.8 [125.7,221.3] |  | 0.216^a^ |
| Lactate, median [Q1,Q3] | 1.9 [1.1,3.3] |  | 1.9 [1.1,3.2] |  | 2.3 [1.3,3.8] |  | <0.001^a^ |
| WBC, median [Q1,Q3] | 10300.0 [6900.0,14800.0] |  | 10375.0 [7100.0,14700.0] |  | 10295.0 [6400.0,15100.0] |  | 0.056^a^ |
| Eosinophil (%), median [Q1,Q3] | 0.1 [0.0,0.5] |  | 0.1 [0.0,0.5] |  | 0.1 [0.0,0.5] |  | 0.45^a^ |
| Basophil (%), median [Q1,Q3] | 0.1 [0.0,0.2] |  | 0.1 [0.0,0.2] |  | 0.0 [0.0,0.2] |  | <0.001^a^ |
| **Ventilation Settings** | | | | | | | |
| Peak Airway Pressure, median [Q1,Q3] | 24.0 [20.3,28.0] |  | 24.0 [20.0,28.0] |  | 26.0 [22.0,29.7] |  | <0.001^a^ |
| Mean Airway Pressure, median [Q1,Q3] | 9.5 [8.0,12.0] |  | 9.0 [8.0,11.5] |  | 10.5 [8.7,13.5] |  | <0.001^a^ |
| FiO_2_, median [Q1,Q3] | 43.3 [35.0,57.9] |  | 41.4 [34.2,55.2] |  | 49.6 [39.6,65.8] |  | <0.001^a^ |

^a^ Kruskal-Wallis test

**Table S4. Performances of HSI model, shock index and systolic blood pressure in TPEVGH cohort**

| **Model** | **AUROC** | **AUROC 95% CI** | **Recall** | **F1** | **Specificity** |
| --- | --- | --- | --- | --- | --- |
| HSI | 0.76 | [0.748, 0.765] | 0.72 | 0.46 | 0.67 |
| Shock Index | 0.7 | [0.686, 0.707] | 0.49 | 0.42 | 0.80 |
| Systolic Blood Pressure | 0.69 | [0.680, 0.702] | 0.37 | 0.39 | 0.88 |

**Table S5. Confusion matrix of HSI model under different threshold**

| **Threshold** | **True Negative** | **False Positive** | **False Negative** | **True Positive** | **Recall** | **F_1** | **Specificity** | **PPV** | **NPV** |
| --- | --- | --- | --- | --- | --- | --- | --- | --- | --- |
| 0 | 0 | 12914 | 0 | 3053 | 1.000 | 0.321 | 0.000 | 0.191 | 1.000 |
| 0.05 | 465 | 12449 | 3 | 3050 | 0.999 | 0.329 | 0.036 | 0.197 | 0.994 |
| 0.1 | 1528 | 11386 | 17 | 3036 | 0.994 | 0.347 | 0.118 | 0.211 | 0.989 |
| 0.15 | 2343 | 10571 | 49 | 3004 | 0.984 | 0.361 | 0.181 | 0.221 | 0.980 |
| 0.2 | 3144 | 9770 | 93 | 2960 | 0.970 | 0.375 | 0.243 | 0.233 | 0.971 |
| 0.25 | 3803 | 9111 | 148 | 2905 | 0.952 | 0.386 | 0.294 | 0.242 | 0.963 |
| 0.3 | 4453 | 8461 | 204 | 2849 | 0.933 | 0.397 | 0.345 | 0.252 | 0.956 |
| 0.35 | 5046 | 7868 | 269 | 2784 | 0.912 | 0.406 | 0.391 | 0.261 | 0.949 |
| 0.4 | 5569 | 7345 | 340 | 2713 | 0.889 | 0.414 | 0.431 | 0.270 | 0.942 |
| 0.45 | 6114 | 6800 | 399 | 2654 | 0.869 | 0.424 | 0.473 | 0.281 | 0.939 |
| 0.5 | 6615 | 6299 | 467 | 2586 | 0.847 | 0.433 | 0.512 | 0.291 | 0.934 |
| 0.55 | 7135 | 5779 | 554 | 2499 | 0.819 | 0.441 | 0.553 | 0.302 | 0.928 |
| 0.6 | 7624 | 5290 | 647 | 2406 | 0.788 | 0.448 | 0.590 | 0.313 | 0.922 |
| 0.65 | 8142 | 4772 | 744 | 2309 | 0.756 | 0.456 | 0.630 | 0.326 | 0.916 |
| 0.7 | 8625 | 4289 | 863 | 2190 | 0.717 | 0.460 | 0.668 | 0.338 | 0.909 |
| 0.75 | 9211 | 3703 | 1014 | 2039 | 0.668 | 0.464 | 0.713 | 0.355 | 0.901 |
| 0.8 | 9781 | 3133 | 1194 | 1859 | 0.609 | 0.462 | 0.757 | 0.372 | 0.891 |
| 0.85 | 10383 | 2531 | 1416 | 1637 | 0.536 | 0.453 | 0.804 | 0.393 | 0.880 |
| 0.9 | 11133 | 1781 | 1761 | 1292 | 0.423 | 0.422 | 0.862 | 0.420 | 0.863 |
| 0.95 | 12106 | 808 | 2336 | 717 | 0.235 | 0.313 | 0.937 | 0.470 | 0.838 |
| 1 | 12914 | 0 | 3053 | 0 | 0.000 | 0.000 | 1.000 | 1.000 | 0.809 |

**Fig. S2. Calibration plot of Hemodynamic Stability Index (HSI) on TPEVGH cohort**


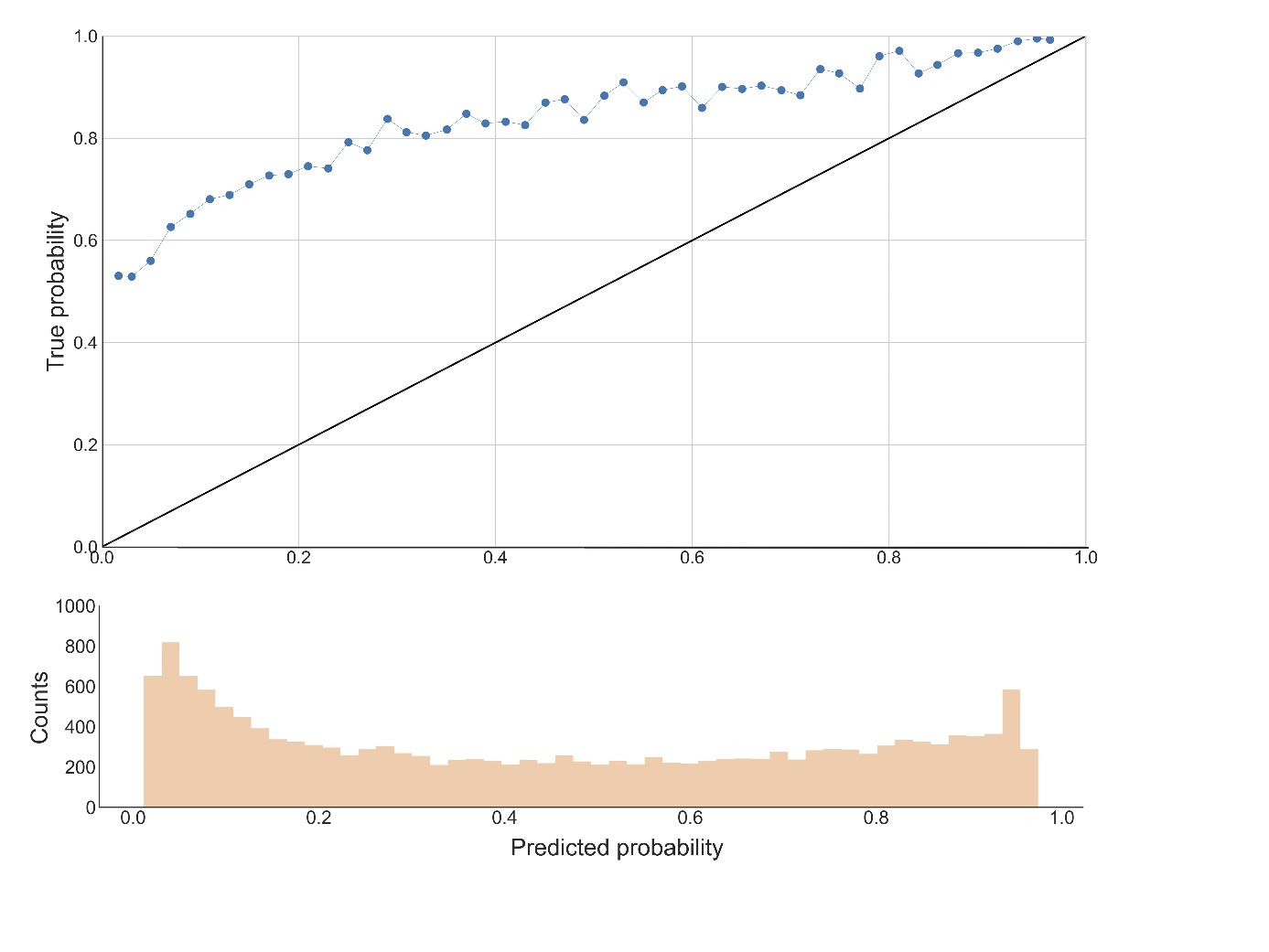


**Fig. S3. False alarm rate for HSI, shock index and Systolic BP**


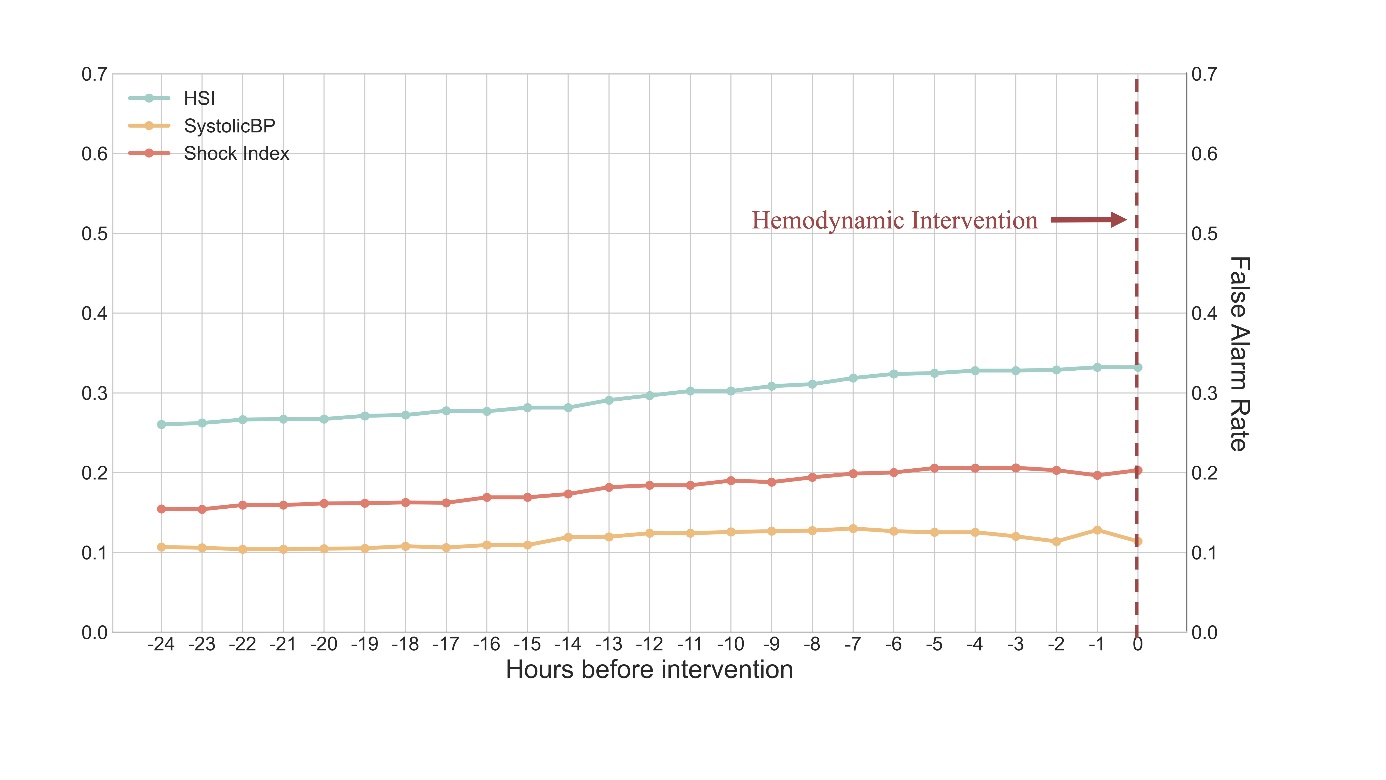


**Table S6. Subgroup performance of HSI model in TPEVGH**

| **Subgroup of Admission Source** | **AUC** | **95% AUC CI** |
| --- | --- | --- |
| Cardiovascular | 0.89 | [0.87, 0.92] |
| Respiratory | 0.74 | [0.56, 0.92] |
| Gastrointestinal | 0.75 | [0.73, 0.77] |
| Neurologic | 0.76 | [0.58, 0.94] |
| Metabolic/Endocrinology | 0.77 | [0.73, 0.80] |
| Trauma | 0.78 | [0.74, 0.83] |
| Others | 0.72 | [0.71, 0.74] |

**Table S7. Median comparison between the US cohort and TPEVGH cohort**

| **Feature** | **TPEVGH Cohort Median** | **United States Cohort Median** |
| --- | --- | --- |
| Age | 70 | 64 |
| Diastolic blood pressure | 63.9 | 65 |
| Systolic blood pressure | 128.6 | 122 |
| Mean blood pressure | 85.4 | 80 |
| Central venous pressure | 9.5 | 10 |
| Heart rate | 89.6 | 81 |
| Temperature | 98.6 | 98.24 |
| Creatinine | 1.5 | 0.9 |
| Blood Urea Nitrogen | 31 | 18 |
| Total Bilirubin | 0.9 | 0.6 |
| Potassium | 3.9 | 4 |
| Sodium | 139 | 139 |
| Calcium | 7.8 | 8.4 |
| SaO_2_ | 99.2 | 97 |
| PaCO_2_ | 29.4 | 40 |
| Base Excess | -2.9 | 0 |
| CO_2_ | 20.1 | 25.6 |
| Hemoglobin | 9.7 | 11 |
| Hematocrit | 28.9 | 33.4 |
| Magnesium | 2 | 2 |
| Partial thromboplastin time | 34 | 32.1 |
| Aspartate transaminase | 47 | 27 |
| Ionized_Calcium | 4.4 | 4.56 |
| Bands | 0 | 5 |
| Glucose | 168 | 123 |
| Lactate | 2 | 1.4 |
| WBC | 10.8 | 9.9 |
| Eosinophils | 0 | 1 |
| Basophils | 0.1 | 0 |
| Peak Airway Pressure | 22.5 | 21 |
| Mean Airway Pressure | 9.4 | 9.4 |
| FiO_2_ | 0.357 | 0.35 |
